# Causes of fever in Tanzanian adults attending outpatient clinics: a prospective cohort study

**DOI:** 10.1016/j.cmi.2020.08.031

**Published:** 2021-06

**Authors:** N. Boillat-Blanco, Z. Mbarack, J. Samaka, T. Mlaganile, T. Kazimoto, A. Mamin, B. Genton, L. Kaiser, V. D'Acremont

**Affiliations:** 1) Ifakara Health Institute, Dar Es Salaam, United Republic of Tanzania; 2) Swiss Tropical and Public Health Institute, University of Basel, Basel, Switzerland; 3) Infectious Diseases Service, University Hospital of Lausanne, Lausanne, Switzerland; 4) Mwananyamala Hospital, Dar Es Salaam, United Republic of Tanzania; 5) Division of Infectious Diseases and Centre for Emerging Viral Diseases, University of Geneva Hospitals, And Faculty of Medicine, Geneva, Switzerland; 6) Centre for Primary Care and Public Health, University of Lausanne, Lausanne, Switzerland

**Keywords:** Aetiologies of fever, Dengue, Human immunodeficiency virus, Outbreak, Sub-Saharan Africa

## Abstract

**Objectives:**

Exploring fever aetiologies improves patient management. Most febrile adults are outpatients, but all previous studies were conducted in inpatients. This study describes the spectrum of diseases in adults attending outpatient clinics in urban Tanzania.

**Methods:**

We recruited consecutive adults with temperature ≥38°C in a prospective cohort study. We collected medical history and performed a clinical examination. We performed 27 364 microbiological diagnostic tests (rapid tests, serologies, cultures and molecular analyses) for a large range of pathogens on blood and nasopharyngeal samples. We based our diagnosis on predefined clinical and microbiological criteria.

**Results:**

Of 519 individuals, 469 (89%) had a clinically or microbiologically documented infection and 128 (25%) were human immunodeficiency virus (HIV) -infected. We identified 643 diagnoses: 264 (41%) acute respiratory infections (36 (5.6%) pneumonia, 39 (6.1%) tuberculosis), 71 (11%) infections with another focus (31 (4.8%) gastrointestinal, 26 (4.0%) urogenital, 8 (1.2%) central nervous system) and 252 (39%) infections without focus (134 (21%) dengue, 30 (4.7%) malaria, 28 (4.4%) typhoid). Of the 519 individuals, 318 (61%), 179 (34%), 30 (6%) and 15 (3%), respectively, had a viral, bacterial, parasitic and fungal acute infection. HIV-infected individuals had more bacterial infections than HIV-negative (80/122 (66%) versus 100/391 (26%); p < 0.001). Patients with advanced HIV disease had a higher proportion of bacterial infections (55/76 (72%) if CD4 ≤200 cells/mm^3^ and 25/52 (48%) if CD4 >200 cells/mm^3^, p 0.02).

**Conclusions:**

Viral diseases caused most febrile episodes in adults attending outpatient clinics except in HIV-infected patients. HIV status and a low CD4 level strongly determined the need for antibiotics. Systematic HIV screening is essential to appropriately manage febrile patients.

## Introduction

Fever is a frequent condition leading to health-care seeking in low-resource areas, with most patients managed as outpatients [1]. As a result of malaria decline and the improved quality of malaria tests, clinicians face a high number of malaria-negative patients, for whom the cause of fever is unknown [2]. WHO guidelines provide a large differential diagnosis for febrile patients. As laboratory capacity is limited in low-resource settings and clinical presentation is non-specific, clinicians face the challenge of deciding who needs antimicrobial therapy. This absence of guidance results in overuse of antibiotics [3,4]. The spectrum of bacterial, viral, fungal and parasitic agents co-circulating in the community is diverse. There is a need to conduct studies on aetiologies of fever to provide clinicians with an accurate picture of the distribution and prevalence of infections.

Several studies on the causes of fever in adults were conducted in Asia and Africa [[5], [6], [7], [8], [9]]. The majority of them investigated a limited number of aetiologies, included only microbiological data without considering clinical conditions and were performed in inpatients who are usually more severely ill. None used predefined algorithms based on the patients' clinical presentation, and radiological and microbiological results for their final diagnoses.

Identification of the multiple infectious causes of fever is of prime importance to appropriately manage patients. This is the first step to improve guidelines and use antimicrobials rationally [6].

## Materials and methods

### Study design, setting and population

We conducted a prospective cohort study to document fever aetiologies between July 2013 and June 2014 at outpatient clinics of one hospital and three health centres in Dar es Salaam, Tanzania. We screened consecutive adults (age ≥18 years) with fever during working hours. Inclusion criteria were: ≤7 days history of fever, tympanic temperature ≥38.0°C and first consultation for the present problem. Exclusion criteria were: refusal of human immunodeficiency virus (HIV) testing, injury or trauma as main reason for consultation, hospital admission during the preceding month or delivery within the last 6 weeks.

The Ifakara Health Institute Review Board (IHI/IRB/No: 12-2013), the Medical Research Coordinating Committee of the National Institute for Medical Research (NIMR/HQ/R.8a/Vol. IX/1561) of Tanzania and the Ethics Committee of the canton of Basel of Switzerland (Ref. Nr. EK: 1612/13) gave ethical approval. All included patients signed an informed consent form.

Clinicaltrials.gov Identifier: NCT01947075.

### Study procedures

We collected at inclusion information on demographics, co-morbidities, symptoms, and vital and clinical signs using a standardized electronic case report form.

We performed the following microbiological tests in all patients: blood cultures; rapid diagnostic tests for HIV, dengue, malaria, typhoid fever; multiplex PCR targeting tropical causes of fever on whole blood and multiplex PCR for respiratory pathogens on nasopharyngeal swabs. In subgroups of patients using predefined algorithms, we performed additional diagnostic tests (see Supplementary material, Table S1) [8]. We performed chest X-rays in patients with clinical pneumonia [10]. The CLSI guidelines were used for antimicrobial susceptibility testing.

We assessed clinical outcome by a day 28 phone call.

We established the aetiologies of fever based on predefined clinical, radiological and microbiological criteria derived from WHO, IDSA, ESCMID guidelines, reviews and expert opinion papers (see Supplementary material, Table S2) [8]. Where different microbiological tests targeted the same pathogen, their results were combined to minimize over- and under-diagnoses. A computer-based algorithm generated diagnoses based on the criteria described in the Supplementary material (Table S2). The same patient could have several diagnoses. We attributed patients to different categories of syndromes according to their clinical presentation, i.e. respiratory tract infections, fever with other focus (urogenital, gastrointestinal, central nervous system, intra-abdominal, skin and joint) and fever without focus.

### Statistical analysis

We compared final diagnoses, demographic, clinical and laboratory characteristics between HIV-infected and HIV-negative patients (Wilcoxon–Mann–Whitney and χ^2^ tests).

For seasonality, we evaluated the association between the proportion of the most frequent diagnostic groups (dengue, malaria, influenza, radiological pneumonia, tuberculosis and typhoid) and each month (χ^2^ test). We analysed the effect of the dengue outbreak on the temporal evolution of the three clinical syndromes by plotting the predicted probability for each syndrome either with or without dengue patients using linear regression.

All analyses were performed with STATA software (version 13.1, Stata Corp, College Station, TX, USA) and GraphPad Prism 6 (GraphPad, San Diego, CA, USA).

## Results

Of 641 adult patients with fever who were screened, 122/641 (19%) did not fulfil the inclusion criteria and/or met exclusion criteria, leaving a study population of 519 patients (Fig. 1). Median age was 30 years (interquartile range 23–40 years) and 273/519 (53%) were female (Table 1); 128/519 (25%) patients were HIV-infected. Compared with HIV-negative patients, HIV-infected individuals were older (median of 35 versus 27 years; p < 0.001), more often female (81/128 (63%) versus 192/391 (49%); p 0.005), had a lower socio-economic status (p 0.01) and were more frequently underweight (p 0.02). HIV status affected antibiotic prescription (84/128 (66%) in HIV-infected versus 126/391 (32%) in HIV-negative individuals; p < 0.001) and 28-day case fatality rate (18/128 (14%) in HIV-infected versus 14/391 (3.6%) in HIV-negative individuals; p < 0.001). HIV infection was a new diagnosis in the majority (76/128 (59%)) of HIV-infected patients. Six of 128 (4.7%) presented with an acute HIV infection; 76/128 (59%) had advanced disease (CD4^+^ T cells <200 cells/mm^3^).

**Fig. 1:**
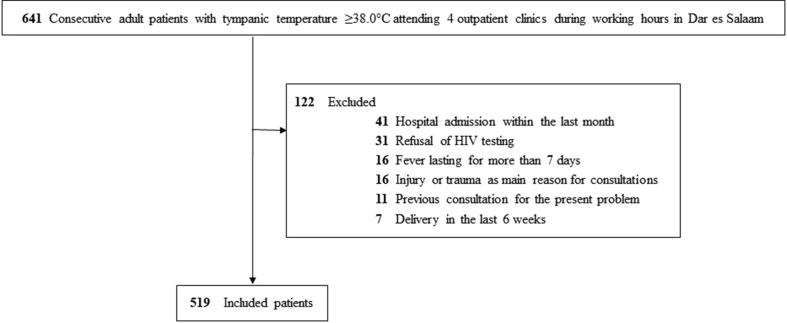
Flow chart of study participants.

**Table 1:** Characteristics of the study population

	All (*n* = 519)	HIV-infected (*n* = 128)	HIV-negative (*n* = 391)	p value
Age (years)	30 (23–40)	35 (29–41)	27 (22–37)	<0.001
Female sex	273 (53)	81 (63)	192 (49)	0.005
Human immunodeficiency virus (HIV)	128 (25)			
Advanced HIV a	76 (59)			
Not previously known for HIV	76 (59)			
Acute HIV infection	6 (4.7)			
Known diabetes	8 (1.6)	1 (0.8)	7 (1.8)	0.44
Pregnancy	12 (2.3)	1 (0.81)	11 (2.82)	0.005
Smoking	37 (7.2)	12 (9.7)	25 (6.4)	0.22
Alcohol misuse	14 (2.7)	5 (4.0)	9 (2.3)	0.30
Past history of tuberculosis	23 (6.6)	12 (18)	11 (3.9)	<0.001
Travel history in the last month	82 (16)	109 (88)	323 (83)	0.18
Present contact with sick persons	22 (4.3)	3 (2.4)	19 (4.9)	0.24
Close contact with cattle in the last month	2 (0.39)	0 (0)	2 (0.5)	0.42
Socioeconomic status				0.01
Low	58 (11)	22 (18)	36 (9.4)	
Medium	271 (54)	67 (55)	204 (53)	
High	177 (35)	33 (27)	144 (38)	
Low body mass index b	110 (23)	36 (30)	74 (20)	0.02
Temperature				0.95
38°C to <39°C	389 (75)	95 (74)	294 (75)	
39°C to <40°C	112 (22)	28 (22)	84 (21)	
≥40°C	18 (3)	5 (3.9)	13 (3.3)	
28-day mortality	32 (6.2)	18 (14)	14 (3.6)	<0.001
Admission	81 (16)	34 (27)	47 (12)	<0.001
Length of hospital stay (days)	4 (2–8)	6 (3–9)	3.5 (2–7)	0.07
Antibiotic prescription	210 (40)	84 (32)	126 (66)	<0.001

Values are given as median (interquartile range) or as *n* (%).

^c^ SOFA score ≥2 points.

a CD4^+^ T-cell count <200 cells/μL.

b Missing data in 31; low body mass index <18.5 kg/m^2^.

### Final diagnoses and types of pathogens

In total, we performed 27 364 microbiological tests and 181 chest X-rays.

Of the 519 enrolled patients, 469 (89%) presented clinical features and/or microbiological results that matched our computer-based decision tree, and 56/519 (11%) remained non-characterized (fever of unknown aetiology). Fig. 2a shows the 643 identified diagnoses; 108/519 (21%) patients had two or more diagnoses (range one to four). The most frequent site of presumed infection was the respiratory tract with 188/643 (29%) lower respiratory tract infections (Figs. 2b) and 76/643 (12%) upper respiratory tract infections. Thirty-nine per cent (252/643) of the diagnoses were microbiologically documented infections without clinical focus (Fig. 2a).

**Fig. 2:**
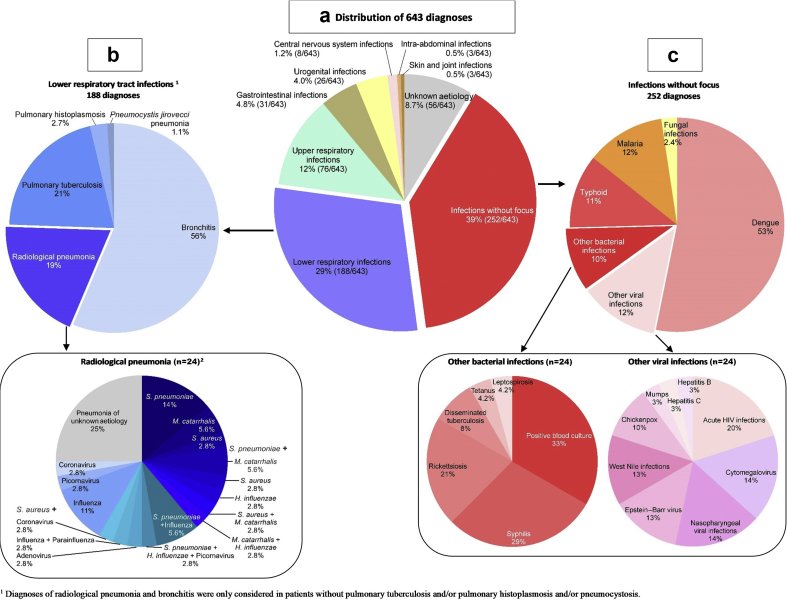
Distribution of 643 diagnoses found in 519 adults attending outpatient clinics with fever in Tanzania. (a) All diagnoses; (b) diagnoses of lower respiratory tract infections; (c) diagnoses of infections without focus.

Fig. 3 shows the distribution of diseases by pathogen type. A viral infection was documented in most patients (318/519 (61%)), a bacterial one in 179/519 (34%), a parasitic infection in 30/519 (6%) and a fungal infection in 15/519 (3%); a single pathogen type was documented in 385/519 (74%) of the patients, two pathogen types in 77/519 (15%), and three pathogen types in one patient.

**Fig. 3:**
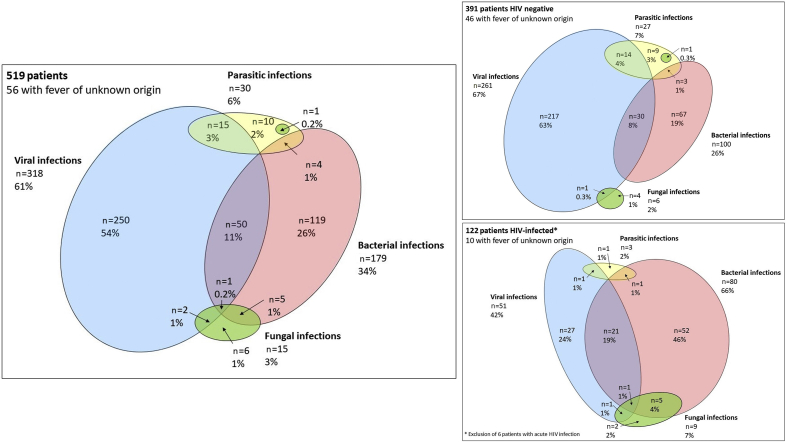
Overlap between types of pathogens causing fever in the 519 adults, overall and stratified by HIV status.

#### Viral diseases

Dengue was the most common viral disease, identified in 134/318 patients (42% of viral infections). Dengue was seasonal and detected during an outbreak between January and June 2014. We detected other viruses in the blood of 19 patients (four Epstein–Barr virus, five cytomegalovirus, four West Nile virus and six acute HIV infection). Three patients were diagnosed with chickenpox and one with mumps.

Among 263 patients with an acute respiratory tract infection, 82/263 (31%) had a respiratory RNA virus detected in the nasopharyngeal swab: 29% (22/76) of those presenting with an upper respiratory tract infection, 41% (43/106) of those with bronchitis and 36% (13/36) of those with pneumonia (Fig. 2b).

We detected viruses in the stool of 6/31 (19%) patients with gastroenteritis (four adenovirus, two norovirus). Among 19 patients with laboratory hepatitis (three-fold increase in alanine aminotransferase), we diagnosed one with hepatitis B and one with hepatitis C (no hepatitis A).

#### Bacterial diseases

Among the 519 patients, 23/519 (4.4%) had a bacteraemia (11 *Salmonella typhi*, 4 *Streptococcus pneumoniae,* and 1 of each of the following: *Escherichia coli*, *Moraxella catarrhalis*, *Pseudomonas fluorescens*, *Staphylococcus aureus*, *Salmonella paratyphi*, *Streptococcus pyogenes*, *Shigella sonnei*, *Clostridium* sp.).

Among patients with blood-borne bacterial infections (usually presenting as fever without clinical focus identified), 28 patients had typhoid fever (*Salmonella* in blood/stool culture and/or positive rapid diagnostic test), 7 syphilis, 5 rickettsiosis, 2 disseminated tuberculosis, 1 leptospirosis and 1 tetanus (Fig. 2c).

Among patients with pharyngitis, we identified streptococcal pharyngitis in 13/44 (30%). Among patients with lower respiratory tract infections, we identified tuberculosis in 37/187 (20%) (microbiologically documented in 34/37).

Among 31 gastrointestinal infections, we identified a bacterial pathogen in 23 (in stool: 15 enteroinvasive *E. coli*, 4 *Campylobacter jejuni*, 3 verocytotoxigenic *E. coli*, 1 *Salmonella* sp., 1 *S. paratyphi*, 1 *Vibrio parahaemolyticus*, 1 *Clostridium difficile*). Among 26 genito-urinary tract infections, 24 were urinary tract infection (in urine and/or blood: 12 *E. coli*, 3 *Salmonella* sp., 2 *Klebsiella pneumoniae*, 2 *Serratia odorifera*, 1 *Enterococcus faecium*, 1 *Klebsiella* sp., 1 *Kluyvera* sp., 1 *Ochrobactrum anthropi*, 1 *Proteus vulgaris*), 2 pelvic inflammatory disease (1 *Chlamydia trachomatis*, 1 *Neisseria gonorrhoeae*) and 1 urethritis (*C. trachomatis*).

All bacteria identified were tested for antibiotic susceptibility*—*4/26 (15%) of *E. coli* were resistant to ciprofloxacin, 23/26 (88%) to cotrimoxazole and 3/26 (12%) to third-generation cephalosporins. All *S. typhi* (*n =* 13) were sensitive to ciprofloxacin.

#### Parasitic diseases

We identified parasitic disease in 30/519 patients (5.7%) and all were malaria.

#### Fungal diseases

We identified fungal disease in 15 patients: 7 histoplasmosis (5 pulmonary and 2 without a clinical focus), 6 cryptococcosis (2 meningitis and 4 without a clinical focus) and 2 pneumocystosis.

### Diagnoses according to HIV status

The 159 distinct diagnoses identified among 122 HIV-infected patients and the 473 diagnoses identified among 391 HIV-negative patients are shown in the Supplementary material (Fig. s1). HIV-infected patients more often had a diagnosis of acute respiratory tract infection than HIV-negative individuals (83/159 (52%) versus 179/473 (38%); p 0.002), as well as a diagnosis of gastrointestinal infection (13/159 (8.2%) versus 17/473 (3.6%); p 0.03).

HIV status affected the distribution of the pathogen types. The proportion of patients with a bacterial and a fungal infection was higher among HIV-infected individuals compared with HIV-negative individuals (80/122 (66%) versus 100/391 (26%); p < 0.001 and 9/122 (7.0%) versus 6/391 (1.5%); p 0.003, respectively). The proportion of patients with a viral and a parasitic infection was lower in HIV-infected compared with HIV-negative patients (51/122 (42%) versus 261/391 (67%); p < 0.001 and 3/122 (2.5%) versus 27/391 (6.9%); p 0.01, respectively) (Fig. 3). Patients with advanced HIV disease had a higher proportion of bacterial infections (55/76 (72%) in those with CD4 ≤200 cells/mm^3^ and 25/52 (48%) in those with CD4 >200 cells/mm^3^, p 0.02). All fungal infections were found in patients with advanced HIV disease.

Among patients with microbiologically documented infections without focus, dengue was less prevalent in HIV-infected individuals (9/41 patients (22%) versus 125/194 (64%); p < 0.001). Among patients with lower respiratory tract infections, HIV-infected patients presented more often with tuberculosis (25/73 (34%) versus 14/114 (12%); p < 0.001) and pneumonia (22/73 (30%) versus 14/114 (12%); p 0.004) and less often with bronchitis (24/73 (33%) versus 82/114 (72%); p < 0.001) (see Supplementary material, Fig. S1).

### Seasonality of infections

**Fig. 4:**
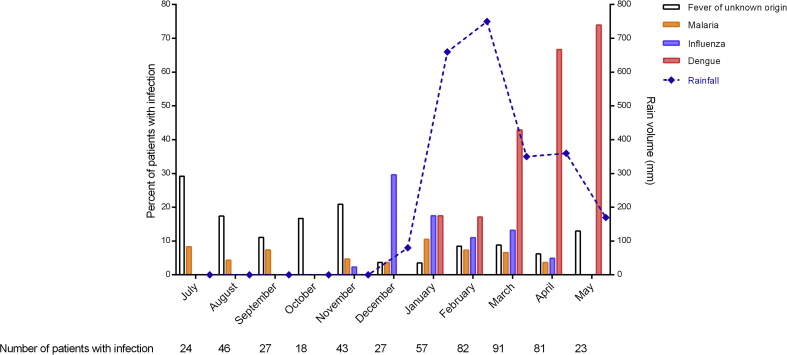
Proportion of patients diagnosed with malaria, dengue, influenza and fever of unknown origin over time. Representation of the rainy season by the volume of rain during the study period.

In December 2013, an outbreak of influenza started and lasted until April 2014. Following heavy rainfall, an outbreak of dengue started in January 2014 peaking from March to June (Fig. 4). We did not observe a pattern of seasonality for malaria, typhoid, pneumonia and tuberculosis. These outbreaks modified the distribution of the clinical syndromes. During the influenza outbreak, the proportion of individuals with acute respiratory tract infection increased (21/43 (49%) in November versus 22/27 (81%) in December; p 0.01). The proportion of patients with fever without focus started to be significantly different in March (47/91 (52%), 95% CI 41%–62%)—i.e. 2 months after detection of the outbreak based on Dengue rapid diagnostic test results—compared with the average rate of previous months (100/324 (31%), 95% CI 26%–36%) (see Supplementary material, Fig. S2).

There was a lower proportion of viral infections before than during the dengue outbreak (80/185 (43%) versus 238/334 (71%); p < 0.001) (see Supplementary material, Fig. S3). After excluding dengue patients, we still identified a viral infection in 184/385 (48%) of patients (136/266 (51%) in HIV-negative and 48/119 (40%) in HIV-infected).

## Discussion

Using a comprehensive algorithm-based work-up, we identified a clinically or microbiologically documented infection in 469/519 (89%) febrile adults in a metropolitan area of Tanzania. For the first time in febrile adults attending outpatient clinics, we show that viral diseases are the main cause of fever. Similar findings were shown in outpatient children [8].

Host and environmental factors affected the distribution of the causes of fever.

In terms of host factors, and in contrast to children in the same setting, the prevalence of HIV infection was higher (25%) than in the general population (7%) [11]. This is expected as immunosuppressed individuals are at higher risk of symptomatic infections. Most HIV-infected individuals had advanced disease (CD4 <200 cells/mm^3^) and a newly diagnosed HIV infection. Bacterial diseases, primarily pneumonia and tuberculosis, affected the majority of HIV-infected individuals in all CD4 cell count strata, contrasting with HIV-negative individuals who mostly presented with viral diseases. Our results are consistent with a study identifying bacterial diseases as the leading cause of fever in HIV-infected patients in Côte d’Ivoire [12].

HIV status should guide diagnostic work-up, especially tuberculosis screening as recommended by WHO [4,13]. It should also guide patient management, particularly in settings with limited microbiological capacities where patients with advanced HIV disease might benefit from receiving an antibiotic presumptively.

In terms of environmental factors, there was a seasonal influence on the distribution of aetiologies of fever. During the rainy season, an outbreak of dengue shifted the distribution of fever aetiologies towards viral diseases. It also modified the distribution of clinical syndromes, patients presenting with fever without focus becoming more prevalent. There was a 2-month delay after the start of the outbreak in the change in the distribution of clinical syndromes, which might be explained by the overlap between outbreaks causing different clinical syndromes (influenza and dengue). Sentinel sites with microbiological capacities to identify unexpected outbreaks should complement syndromic surveillance systems.

In contrast to previous studies in admitted febrile adults in rural Asia and northern Tanzania [7,9], bacterial zoonoses were rare in our population. This might be due to the urban setting of our study [14]. This finding requires confirmation using reference tests (paired serological assays).

We conducted our study in an urban setting of Tanzania and our results can probably be generalized to other similar cities in sub-Saharan countries but not to rural settings. Some microbiological screening faced limitations linked to the performances of the tests. We performed most tests in a targeted fashion following a predefined algorithm based on clinical symptoms and signs, HIV status and CD4 T-cell count, to increase pre-test probability and reduce misinterpretation. We also combined the results of different tests targeting the same organisms to reduce the post-test probability of a false-positive result. Despite these precautions, some diseases are possibly under-diagnosed (zoonoses: insensitive multiplex PCR in blood) and others over-diagnosed (typhoid: limited specificity of rapid diagnostic test; cytomegalovirus/Epstein–Barr virus: non-specific serology). We used PCRs in respiratory samples to diagnose viral/bacterial pneumonia, and viral nasopharyngeal infections. In the absence of a control group, we cannot ensure the causality between pathogen and fever. However, it does not explain the high proportion of viral illnesses, as it concerned 11 individuals.

Some diagnoses relied on clinical criteria only and were categorized in different aetiological categories based on existing scientific knowledge. In case of multiple diagnoses in the same patient, we did not attribute causality to the fever, as we wanted to avoid ‘expert opinion’, which is not reproducible and can be a source of bias.

Our results show for the first time a comprehensive pattern of the causes of fever among adults attending outpatient clinics in urban sub-Saharan Africa. Our findings reinforce WHO recommendations of systematic HIV testing at outpatient clinics in areas of high prevalence and indicate that HIV screening in febrile patients is essential as it affects the probability of bacterial infection and the need for antibiotics.

Evidence-based electronic decision-support algorithms using clinical data, co-morbidities, epidemiological information as well as carefully selected point-of-care tests can be developed using these results [15,16].

## Transparency declaration

The authors declare that they have no competing interests.

## Funding

This work was supported by the 10.13039/100000865Bill and Melinda Gates Foundation (grant OPP-1022128; to VD). This work was also supported by a postdoctoral fellowship of the 10.13039/501100006387Leenaards Foundation (to NBB). The funding bodies had no role in the design of the study and collection, analysis and interpretation of data and in writing the manuscript.

## Availability of data and material

All data files are available at ZENODO (https://zenodo.org/deposit/3241957).

## Authors' contributions

NBB, VDA and BG contributed to study conception, study design, study performance, study management, data analysis, data interpretation and manuscript writing TK, AM and LK contributed to laboratory analysis, data interpretation and critical review of the manuscript. ZM, SJ and TM contributed to acquisition of the data, interpretation of the data and critical review of the manuscript. All authors approved the final version of the manuscript and agreed to be accountable for all aspects of the work in ensuring that questions related to the accuracy or integrity of any part of the work are appropriately investigated and resolved. NBB had full access to all the data in the study and takes responsibility for the integrity of the data and the accuracy of the data analysis.

## Figures and Tables

**figs1:**
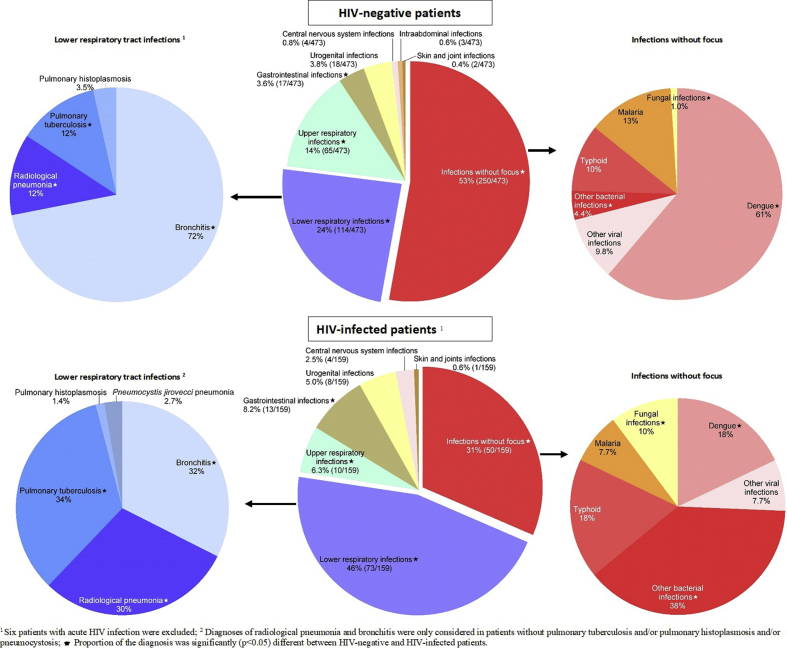
Distribution of diagnoses found in 391 HIV-negative and 128 HIV-infected febrile adults attending outpatient clinics in Tanzania.

**figs2:**
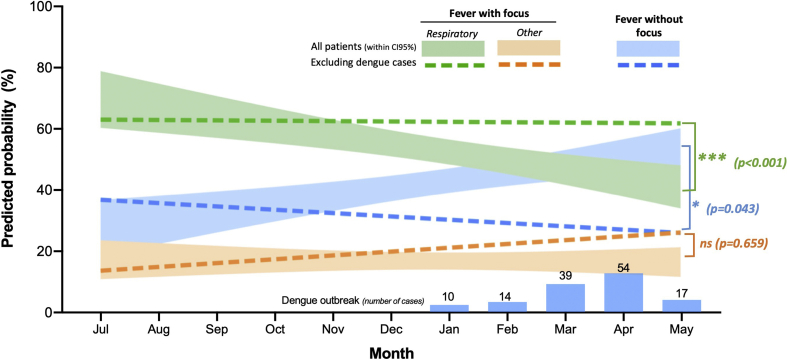
Predicted linear temporal distribution of the major clinical syndromes (with and without dengue patients).

**figs3:**
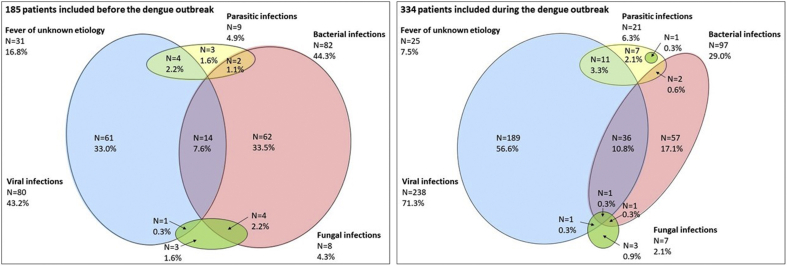
Overlap between types of pathogens before and during the dengue outbreak.
