# Supplementary material for: Causes of fever in Tanzanian adults attending outpatient clinics: a prospective cohort study
Source: Clin Microbiol Infect. 2021 Jun;27(6):913.e1–7. doi: 10.1016/j.cmi.2020.08.031 (PMC8186429; doi:10.1016/j.cmi.2020.08.031)
Supplement: Multimedia component 1 [file mmc1.docx]

Supplementary Table 1. Diagnostic tests performed in the included patients

| **Tests performed in all patients (519/519 patients)** | | |
| --- | --- | --- |
|  | Complete blood count (Horiba Medical ABX Pentra 80)  Serum alanine transferase and serum creatinine (Biochemical Systems International)  Bilirubin (Biochemical Systems International) | |
|  | HIV rapid diagnostic test (Alere Determine^TM^ HIV-1/2, confirmed by Trinity Biotech Uni-gold^TM^ Recombigen^®^ HIV-1/2) | |
|  | Malaria rapid diagnostic test (ICT Malaria P.f.^®^) and if positive, blood slide | |
|  | Typhoid rapid diagnostic test (Reszon Diagnostics International TYPHIDOT Rapid IgM^®^) | |
|  | Blood cultures | |
|  | Real-time multiplex PCR for tropical pathogens in blood (Fast-track DIAGNOSTICS tropical fever core^®^ (ref FTD-3632) and Africa^®^ (ref FTD-3732)) ^1^ | |
|  | Real-time multiplex PCR for respiratory pathogens in nasopharyngeal sample (Fast-track DIAGNOSTICS respiratory pathogens 33^®^ (ref FTD-2P.3-64)) ^2^ | |
|  |  | |
| Additional investigations in patients with **cough and/or dyspnea, and at least 1 symptom/sign among tachypnea (resp rate ≥20/min), pleuritic chest pain or abnormal chest auscultation** | | |
|  | Chest X-ray (performed in 181 patients) | |
|  |  | |
| Additional investigations in patients with **cough and/or dyspnea lasting ≥ 2 weeks or in patients with cough and/or dyspnea and a chest X-ray suggestive of tuberculosis or in HIV infected patients with cough and/or dyspnea of any duration** | | |
|  | TB screening: 2 sputa for GeneXpert^®^ MTB/RIF (performed in 98 patients) | |
|  | Histoplasma antigen in urine and Histoplasma IgM in serum (MiraVista Diagnostics^®^) (performed in 89 patients) | |
|  |  | |
| Additional investigations in patients with **cough or dyspnea infected with HIV and CD4 count <250 cells/mm^3^** | | |
| Immunofluorescence (Fungi-Fluor^®^ kit) and *Pneumocystis jiroveci* PCR of induced sputum (performed in 41 patients) | |  |
| Serum β-D-glucan (MiraVista^®^ Diagnostics) (performed in 185 patients) | |  |
|  | |  |
| Additional investigations in patients **with a negative HIV rapid test** | | |
|  | Serum p24 antigen (performed in 397 patients) | |
|  | | |
| Additional investigations in patients **infected with HIV** **and CD4 count <150 cells/mm^3^** | | |
|  | Serum Cryptococcus antigen (IMMY CrAg^®^) (performed in 90 patients) | |
|  | Alere Determine^®^ TB LAM Ag in urine (performed in 65 patients) | |
|  |  | |
| Additional investigations in patients with **diarrhea** (≥ 3 loose or liquid stools) | | |
|  | Stool examination for *Entamoeba* spp (performed in 17 patients) | |
|  | Stool culture for *Salmonella*, *Campylobacter* and *Shigella* (performed in 23 patients) | |
|  | Real-time multiplex PCR for gastro-intestinal pathogens in stool stool (Fast-track DIAGNOSTICS viral and bacterial gastroenteritis^®^ (ref FTD-3-24/4 and FTD-14-24/4) ^3^ (performed in 33 patients) | |
|  | Rapid test for rotavirus and norovirus in stool (VIKIA Rota-Adeno^®^) (performed in 17 patients) | |
|  |  | |
| Additional investigations in patients with **tonsillitis** | | |
|  | *Streptococcus pyogenes* rapid test (Chemtrue^®^) (performed in 90 patients) | |
|  |  | |
| Additional investigations in patients with **neck stiffness** | | |
|  | Cerebrospinal fluid analyses and culture (performed in 6 patients) | |
|  |  | |
| Additional investigations in patients with **dysuria and/or polyuria** | | |
|  | Urine dipstick and, if positive for leucocytes or nitrites, urine culture (performed in 67 patients) | |
|  |  | |
| Additional investigations **in men with** **urethral discharge and/or dysuria and/or scrotal swelling or tenderness without rotation of the testis and history of trauma** | | |
|  | Urine dipstick and, if positive for leucocytes, PCR for *Chlamydia trachomatis* and *Neisseria gonorrhoea* in urine (performed in 25 patients) | |
|  | | |
| Additional investigations in patients with **vesicles and/or ulcers in the genito-anal area** | | |
|  | Herpes simplex virus PCR in a swab and rapid test for syphilis (Onsite Syphilis Ab Combo^®^) (no patient meeting these criteria) | |
|  | | |
| Additional investigations in patients with **anorectal pain with tenesmus and discharge** | | |
|  | *C. trachomatis* and *N. gonorrhoea* PCR on rectal swab (no patient meeting these criteria) | |
|  | | |
| Additional investigations in patients with **genital ulcer and/or maculo-papular rash involving palms and/or soles and/or HIV-infected** | | |
|  | Rapid test for syphilis (Onsite Syphilis Ab Combo^®^) (performed in 129 patients) | |
|  | | |
| Additional investigations in **female with low abdominal tenderness** | | |
|  | Real-time multiplex PCR for genital pathogens in a vaginal swab (Fast-track DIAGNOSTICS vaginal swab^®^ (ref FTD-42-24/4)) ^4^ (performed in 25 patients) | |
|  | | |
| Additional investigations in patients with a **vesicular rash** | | |
|  | Herpes zoster virus PCR in a vesicle swab (Fast-track DIAGNOSTICS vesicular rash^®^) ^5^ (performed in 6 patients) | |
|  | | |
| Additional investigations in patients **with elevated liver enzymes (3-fold increase in ALT) and without definitive diagnosis at inclusion** | | |
|  | Serologies for hepatitis A (IgM), B (hepatitis B HBs antigen and HBc IgM), C (Ig) (performed in 17 patients) | |
|  | | |
| Additional investigations in patients **without definitive diagnosis at inclusion** | | |
|  | Serologies for Ebstein Barr virus (IgM), Cytomegalovirus (IgM), *Toxoplasma gondii (IgM)* | |
|  | Histoplasma antigen in urine and Histoplasma IgM in serum (MiraVista Diagnostics^®^) | |
|  | PCR for Rift Valley fever (in-house designed PCR) (performed in 86 patients) | |
|  |  | |

^1^ PCR targeting dengue virus, chikungunya virus, West Nile virus, *Plasmodium* spp, *Rickettsia* spp, *Leptospira* spp, *Salmonella* spp, *Brucella* spp, yellow fever virus, *Streptococcus pneumoniae* and *Coxiella burnetii*

^2^ PCR targeting bacteria (*Streptococcus pneumoniae*, *Haemophilus influenzae, Haemophilus influenzae type B*, *Moraxella catarrhalis*, *Mycoplasma pneumoniae*, *Bordetella pertussis, Staphylococcus aureus,*  *Legionella pneumophila/longbeachae, Chlamydia pneumonia, Klebsiella pneumonia, Salmonella spp.), a fungus (Pneumocystis jirovecii)* and viruses *(*influenzaA/B/C, picornavirus (rhinovirus), RSV A/B, adenovirus, CMV, parainfluenzae 1-4, coronavirus HKU1, 229E, OC43 and NL63, bocavirus, human metapneumovirus A/B, parechovirus or enterovirus

^3^ PCR targeting norovirus, astrovirus, rotavirus, adenovirus, sapovirus, *Salmonella* spp, *Shigella* spp/EIEC, *Yersinia enterocolitica*, *Clostridium difficile*, *Campylobacter coli/jejuni*; VTEC

^4^ PCR targeting *Chlamydia trachomatis*, *Neisseria gonorrhoeae*, *Mycoplasma genitalium*, *Trichomonas vaginalis, Ureaplasma urealyticum, Ureaplasma parvum and Mycoplasma hominis,* herpes simplex virus 1, 2

^5^ PCR targeting herpes simplex 1, 2, varicella-zoster virus

# Supplementary Table 2. Pre-defined clinical and laboratory criteria for computer-based diagnoses. Bacterial diseases are written in red, viral diseases in green, fungal diseases in blue and parasitic diseases in orange

| **Diagnosis** | **Clinical presentation considered for diagnosis** | | **Basic criteria for a documented diagnosis (usually clinical)** | **Additional requested criteria for a documented diagnosis (usually laboratory test or radiology)** | | |  |
| --- | --- | --- | --- | --- | --- | --- | --- |
|  | | | | | | |  |
| **ACUTE RESPIRATORY INFECTIONS** | | | | | | |  |
| **LOWER RESPIRATORY TRACT INFECTIONS** | | | | | | |  |
| Diagnoses of radiological pneumonia, COPD exacerbation and bronchitis were only considered in patients without pulmonary tuberculosis and/or pulmonary histoplasmosis and/or *Pneumocystis jirovecii* pneumonia | | | | | | |  |
| Pulmonary tuberculosis | Cough and/or dyspnea | | - Cough>2 weeks - In HIV infected patients: cough of any duration - Cough and Xray suggestive of tuberculosis | - Positive GeneXpert MTB/RIF in sputum - If HIV infected and CD4 count <150 cells/mm^3^: positive TB LAM Ag in urine - If xray suggestive of TB: decision by the medical doctor in charge to treat with a full course of treatment and absence of pulmonary histoplasmosis | | |  |
| Pulmonary histoplasmosis | Cough and/or dyspnea | | - Cough>2 weeks - In HIV infected patients: cough of any duration | - Positive Histoplasma antigen in urine - Positive Histoplasma IgM in serum | | |  |
| *Pneumocystis jirovecii* pneumonia | Cough and/or dyspnea | | - HIV infected patients with CD4 count <250 cells/mm^3^ | - Pcp cysts/trophozoites by immunofluorescence of an induced sputum - β-D-glucan ≥80 AND positive PCR for Pcp in an induced sputum or nasopharyngeal swab | | |  |
| Bacterial radiological pneumonia | Cough and/or dyspnea | | - Fast breathing (RR≥20/min) OR pleuritic chest pain OR abnormal chest auscultation | Infiltrate on chest Xray likely to be new | | - Positive PCR for a respiratory bacteria^1^ on nasopharyngeal swab (cycle threshold ≥ 35) - No microorganism identification |  |
| Viral radiological pneumonia |  |  |  |  |  | - Positive PCR for a respiratory virus^2^ on nasopharyngeal swab |  |
| COPD exacerbation | Cough and/or dyspnea | | - COPD - Acute worsening of COPD symptoms (shortness of breath, quantity and color of phlegm) | Absence of infiltrate on chest Xray | | |  |
| Bronchitis | Cough and/or dyspnea | | - Fast breathing (RR≥20/min) OR pleuritic chest pain OR abnormal chest auscultation | Absence of infiltrate on chest Xray | | |  |
| **UPPER RESPIRATORY TRACT INFECTIONS (only in patients without a lower respiratory tract infection)** | | | | | | |  |
| For patients with more than one acute respiratory infection, only one diagnosis was retained in the following order, considering first lower respiratory tract infections and second upper respiratory tract infections.:  Mastoiditis > Tonsillitis > Acute otitis media > Tracheobronchitis > Rhinosinusitis | | | | | | |  |
| Mastoiditis | Swelling and pain behind ear | |  |  | | |  |
| Streptococcal tonsillitis | Throat pain | | - Tonsillar exudate OR tender anterior adenopathy OR absence of cough | Positive RDT for group A streptococcus (GAS) | | |  |
| Non-streptococcal tonsillitis |  |  |  | Negative GAS RDT | | |  |
| Acute otitis media | Ear pain | | - Redness and bulging of tympanic membrane |  | | |  |
| Tracheobronchitis | Cough and/or dyspnea | |  |  | | |  |
| Rhinosinusitis | Rhinitis | | - Rhinitis for less than 8 days |  | | |  |
| **OTHER** **LOCALIZED INFECTIONS** (other than acute respiratory infection) | | | | | | |  |
| **CENTRAL NERVOUS SYSTEM INFECTIONS** | | | | | | |  |
| **Meningitis** | | | | | | |  |
| Bacterial meningitis | | Neck stiffness OR Kernig’ sign OR Brudzinski’s sign | | | Positive CSF culture | |  |
| Viral meningitis | |  |  |  | Negative CSF culture | |  |
| Fungal meningitis | |  |  |  | Positive cryptococcal antigen in serum | |  |
| **Intracranial infection** (brain abscess or encephalitis) | | Localizing neurologic signs | | |  | |  |
| **GASTROINTESTINAL INFECTIONS** | | | | | | |  |
| Amoebic gastroenteritis | Diarrhoea (≥ 3 stools/day) | | | Positive direct stool examination for Amoeba | | |  |
| Bacterial gastroenteritis |  |  |  | Stool culture positive for *Salmonella* non typhi, *Shigella* or *Campylobacter* OR stool PCR positive for *EIEC, VTEC, Clostridium difficile or Salmonella* spp (in patients without a diagnosis of typhoid) | | |  |
| Viral gastroenteritis |  |  |  | Positive RDT for Rotavirus or Adenovirus OR stool PCR positive for Norovirus, Rotavirus or Adenovirus | | |  |
| Gastroenteritis of unknown origin |  |  |  | - Negative direct examination for Amoeba AND negative stool culture AND negative stool PCR AND negative viral RDT - No stool examination performed | | |  |
| **INTRAABDOMINAL INFECTIONS** | | | | | | |  |
| Liver abscess | Liver abscess on ultrasound | | |  | | |  |
| Peritonitis | Abdominal rebound tenderness and muscle guarding on examination | | |  | | |  |
| Cholangitis | Abdominal pain and jaundice | | |  | | |  |
| **UROGENITAL INFECTIONS** | | | | | | |  |
| Urinary tract infection | Dysuria OR pollakiuria OR costovertebral angle tenderness | | | Leukocytes or nitrites on urine dipstick AND positive urine culture (non-contaminant bacteria ≥10^3^ cfu/ml, *Salmonella* spp was only considered in patients without typhoid) | | |  |
| Pelvic Inflammatory disease | Low abdominal tenderness in female | | | Positive PCR for *N. gonorrhea* or *C. trachomatis* on self-taken vaginal swab | | |  |
| Urethritis | Uretral discharge OR scrotal pain in men | | | Positive PCR for *N. gonorrhea* or *C. trachomatis* on urine | | |  |
| Proctitis | Rectal pain and/or rectal discharge | | | Positive PCR for *N. gonorrhea* or *C. trachomatis* on rectal swab | | |  |
| Genital herpes | Genital vesicles and/or ulcers | | | Positive PCR for HSV1/2 on mucosal swab | | |  |
| **SKIN, SOFT TISSUE AND JOINT INFECTIONS** | | | | | | |  |
| Skin infection | Erysipela or cellulitis or skin abscess | | |  | | |  |
| Arthritis | Joint pain and swelling | | |  | | |  |
|  | | | | | | |  |
| **INFECTIONS WITHOUT FOCUS** | | | | | | |  |
| Malaria | All patients | | | Positive malaria RDT (HRP2 or pLDH) or high quality blood smear | | |  |
| Typhoid | All patients | | | Positive typhoid RDT OR blood or stool culture positive for *Salmonella typhi* | | |  |
| Dengue | All patients | | | Positive dengue RDT (NS1 or IgM) OR blood PCR positive for dengue virus | | |  |
| Bloodstream infection without focal infection | All patients | | - Absence of focal infection likely due to the isolated bacterium - Absence of typhoid in case of blood PCR positive for *Salmonella* spp | Blood culture positive for bacteria other than typical contaminants or blood PCR positive for *Salmonella* spp or *S. pneumoniae* | | |  |
| Disseminated tuberculosis | All patients | | If HIV infected and CD4 count <150 cells/mm^3^ | Positive TB LAM Ag in urine | | |  |
| Cryptococcal infection | All patients without meningitis | | If HIV infected and CD4 count <150 cells/mm^3^ | Positive cryptococcal antigen test on blood | | |  |
| Mumps | Enlarged parotids | | | Positive IgM for mumps | | |  |
| Measles | Non-vesicular generalized rash | | | Positive IgM and negative IgG for measles | | |  |
| Chickenpox | Characteristic generalized vesicular rash | | | Positive PCR for Varicella zoster virus on a vesicle swab | | |  |
| Tetanos | Spasms and stiffness in jaw, neck, abdominal and limb muscles with painful body spasms | | |  | | |  |
| Syphilis | HIV infected patients or generalized non-vesicular rash or ulcer in the genito-anal area | | | Positive syphilis RDT | | |  |
| EBV primary infection | No diagnosis at that point: no respiratory and no other localized infection as well as no malaria, dengue, typhoid, bloodstream infection, disseminated tuberculosis, cryptococcal infection, mumps, measles, chickenpox, tetanos or syphilis | | | Positive IgM for EBV | | |  |
| CMV primary infection |  |  |  | Positive IgM for CMV | | |  |
| Toxoplasmosis primary infection |  |  |  | Positive IgM for *Toxoplasma* | | |  |
| Histoplasmosis |  |  |  | Positive Histoplasma antigen in urine  Positive Histoplasma IgM in serum | | |  |
| Rift Valley |  |  |  | PCR blood positive for Rift Valley virus | | |  |
| Rickettsiosis | All patients | | | Positive blood PCR for *Rickettsia* spp | | |  |
| Leptospirosis | All patients | | | Positive blood PCR for *Leptospira* spp | | |  |
| Chikungunya | All patients | | | PCR blood positive for chikungunya virus | | |  |
| West Nile | All patients | | | PCR blood positive for West Nile virus | | |  |
| Brucellosis | All patients | | | PCR blood positive for *Brucella* spp | | |  |
| Q fever | All patients | | | PCR blood positive for *Coxiella burnetii* | | |  |
| Primary HIV infection | Negative HIV screening by antibody RDT | | | Positive serum p24 antigen | | |  |
| Acute hepatitis A | 3-fold increase in ALT | | | Positive IgM for hepatitis A virus | | |  |
| Acute hepatitis B |  |  |  | Positive Hbs antigen for hepatitis B virus | | |  |
| Acute hepatitis C |  |  |  | Positive antibodies against hepatitis C | | |  |
| Nasopharyngeal viral infection | No diagnosis at that point | | | Positive PCR for a virus on the nasopharyngeal swab | | |  |
| Fever of unknown origin | No diagnosis at that point | | |  | | |  |
|  |  | | |  | | |  |

*^1^ Streptococcus pneumoniae, Haemophilus influenzae, Moraxella catarrhalis, Staphylococcus aureus;* *Legionella pneumophila* or *Chlamydia pneumoniae*

^2^ influenza, picornavirus, RSV, adenovirus, parainfluenzae, coronavirus, bocavirus, metapneumovirus, enterovirus

Definitions: RDT=rapid diagnostic test; cfu= colony forming units; PCR=polymerase chain reaction; CSF=cerebrospinal fluid; ALT=alanine aminotransferase; GAS=Group A Streptococcus; CXR= chest X-ray; IgM=immunoglobulin M; IgG=immunoglobulin G; EBV=Epstein-Barr virus; CMV=cytomegalovirus
